# Unraveling the Taxonomic Diversity and Functional Potential of the Tunisian Salterns, Abbassia and Thyna, via Integrated 16S-18S Amplicons and Shotgun Metagenomics

**DOI:** 10.3390/ijms27114714

**Published:** 2026-05-23

**Authors:** Sondes Mechri, Afef Najjari, Séverine Croze, Hadda-Imene Ouzari, Marilize Le Roes-Hill, Slim Tounsi, Joel Lachuer, Bassem Jaouadi

**Affiliations:** 1 Laboratoire des Biotechnologies Microbiennes et Enzymatiques et Biomolécules (LBMEB), Centre de Biotechnologie de Sfax (CBS), Université de Sfax (USF), Route Sidi Mansour Km 6, BP 1177, Sfax 3018, Tunisia; sondes.mechri@yahoo.com; 2 Univ Lyon, Université Claude Bernard Lyon 1 (UCBL), Plateforme ProfileXpert de Génomique et Microgénomique, SFR Santé-Lyon-Est, CNRS UMR-S3453, INSERM US7, Faculté de Pharmacie de Lyon, 8 Avenue Rockefeller, 69373 Lyon, Cedex 08, France; severine.croze@univ-lyon1.fr; 3 Laboratoire Microorganismes et Biomolécules Actives (LMBA), Département de Biologie, Faculté des Sciences de Tunis (FST), Université de Tunis El Manar (UTM), Foyer Universitaire, 20 Rue de Tolède, Tunis 2092, Tunisia; afef.najjari@fst.utm.tn (A.N.); imene.ouzari@fst.utm (H.-I.O.); 4 Applied Microbial and Health Biotechnology Institute (AMHBI), Cape Peninsula University of Technology (CPUT), P.O. Box 1906, Bellville 7535, South Africa; leroesm@cput.ac.za; 5 Laboratoire de Biopesticides (LB), Centre de Biotechnologie de Sfax (CBS), Université de Sfax (USF), Route Sidi Mansour Km 6, BP 1177, Sfax 3018, Tunisia; 6 Univ Lyon, Université Claude Bernard Lyon 1 (UCBL), UMR INSERM 1052, CNRS 5286, Centre Léon Bérard, Centre de Recherche en Cancérologie de Lyon (CRCL), 28 Rue Laennec, 69008 Lyon, Cedex 08, France

**Keywords:** hypersaline environments, Tunisian salterns, multi-platform sequencing, 16S/18S rRNA, shotgun metagenomics, functional profiling, microbial extremophiles, metabolic adaptation

## Abstract

Hypersaline environments are unique ecosystems harboring specialized microbial communities with significant biotechnological potential. This study provides a comprehensive characterization of the taxonomic diversity and functional potential of two Tunisian salterns, Abbassia (Kerkennah) and Thyna (Sfax), using an integrated approach that combines 16S/18S rRNA gene amplicons (Illumina and full-length Nanopore) with shotgun metagenomics. Taxonomic profiling revealed a high species richness (S ≈ 1250 taxa); however, the Abbassia site was characterized by extreme taxonomic polarization, with over 95% of the community dominated by specialized halophilic *Bacillota* (*Salinicoccus* and *Jeotgalicoccus*). In contrast, Thyna exhibited a more even distribution dominated by *Pseudomonadota* and methanogenic *Archaea*. Beyond taxonomy, functional annotation via the HUMAnN 3.0 pipeline identified site-specific metabolic specializations. Abbassia was enriched in biosynthetic pathways and robust stress-response mechanisms, including ectoine biosynthesis and ppGpp-mediated stringent response, reflecting adaptation to stable hypersaline conditions. Conversely, Thyna’s microbiome prioritized energy extraction and nutrient recycling, with a high abundance of fermentation and glyoxylate cycle pathways. These findings demonstrate that environmental filtering shapes not only the microbial structure but also the metabolic landscape, highlighting the ecological plasticity of microbial life in extreme Tunisian salterns.

## 1. Introduction

Extreme hypersaline environments, characterized by salt concentrations significantly exceeding those of seawater (>35 g/L), represent some of the most challenging ecosystems and extreme habitats on Earth [1]. These habitats include saline soils, salt pans, solar salterns, and sebkhas, where life is continuously shaped by severe environmental filters such as hypersalinity, limited nutrient availability, high osmotic pressure, intense solar radiation, and low oxygen solubility. Solar salterns are unique hypersaline ecosystems characterized by a dynamic salt concentration gradient that progresses from seawater salinity to salt saturation in the crystallizer ponds [2].

Despite these harsh conditions, hypersaline environments harbor some of the most remarkable and specialized microbial communities on Earth. Their study provides unique opportunities to explore microbial adaptation, evolution, and biotechnological potential [3]. Microorganisms inhabiting hypersaline systems have evolved diverse physiological and molecular mechanisms to survive under osmotic and oxidative stress [4]. Historically, biological studies of these environments have predominantly focused on the adaptation of prokaryotes to extreme halophilic conditions [5]. However, these habitats are not limited to prokaryotic life; they host a complex multi-domain network where micro-eukaryotes, including halophilic fungi (often termed “black yeasts”) [6] and specialized micro-algae such as *Dunaliella salina*, coexist with dense prokaryotic communities such as halophilic bacteria and archaea [7].

In fact, although prokaryotic populations have dominated hypersaline microbiology research, the diversity and functional roles of eukaryotic microorganisms remain comparatively understudied. While eukaryotic microbes are fundamental drivers of nutrient cycling and food web regulation in saline ecosystems, their taxonomic resolution remains constrained by the limitations of traditional sequencing methods [8]. These eukaryotic organisms are not merely transient inhabitants but active ecological players. For instance, micro-algae serve as primary producers forming the base of the food web, while halophilic fungi (e.g., *Aspergillus* and *Penicillium* species) act as essential decomposers of organic matter [9]. To survive salt saturation, these eukaryotes have evolved sophisticated mechanisms, such as the intracellular accumulation of glycerol as a compatible solute and the synthesis of protective carotenoid pigments. Moreover, their metabolic versatility and resilience to salinity make them valuable sources of novel enzymes, secondary metabolites, and biomolecules of potential industrial and environmental relevance. The advent of metagenomics and high-throughput sequencing technologies has revolutionized the study of microbial ecology [10]. While much is known about the taxonomic distribution of haloarchaea and halophilic bacteria, there is still a significant gap in our understanding of the inter-domain functional synergies and the full metabolic potential of eukaryotic communities. In particular, the secondary metabolites produced by these “extremophiles” to compete or communicate, often encoded by Biosynthetic Gene Clusters (BGCs), represent a largely untapped resource for biotechnology [11,12].

Unlike traditional culture-dependent methods, metagenomic approaches allow direct access to the genetic material of all organisms present in a sample, thereby providing an unbiased and comprehensive view of microbial community structure and function [13]. In this sense, two complementary strategies are commonly employed: shotgun metagenomics, which sequences all genomic DNA fragments to reconstruct metabolic pathways and genomes [14], and amplicon-based metagenomics, which targets specific conserved genes such as the 16S rRNA and 18S rRNA genes, or the internal transcribed spacer (ITS) region for taxonomic profiling [15]. The amplicon metagenomic sequencing is also time- and cost-efficient, allowing the simultaneous analysis of numerous samples without labor-intensive cloning or cultivation steps [16,17,18]. In Tunisia, solar salterns represent ecosystems of particular ecological and economic importance, yet their microbial eukaryotic diversity remains largely unexplored [19].

Among these, the Thyna and Abbassia solar salterns are human-controlled, semi-artificial environments used for the harvesting of salt for human consumption. Salterns represent ecosystems of particular ecological and economic importance, characterized by intense evaporation, seasonal salinity fluctuations, and anthropogenic impacts. Life in industrial solar salterns is governed by poly-extreme stressors, including high osmotic pressure, fluctuating pH, and limited oxygen availability.

Amplicon-based research frequently suffers from primer mismatch and ribosomal RNA gene Copy Number (RCN) variations, which can obscure the true abundance of the archaeal core and result in a notable overrepresentation of particular taxa, such as members of the phylum, *Bacillota* [20,21]. This study uses a hybrid multimodal approach to address these taxonomic distortions, incorporating high-accuracy Oxford Nanopore Technologies (ONT) long-read scaffold capabilities, which are combined with Illumina short reads. By switching from a targeted 16S/18S rRNA perspective to an untargeted shotgun metagenomic landscape, this approach enables a strong triangulation of microbial diversity.

Additionally, we offer a unique comparative framework to understand how local environmental settings and nutrient availability shape the functional and taxonomic assembly of Tunisian hypersaline ecosystems by comparing the more isolated insular system of Abbassia with the mainland coastal saltern of Thyna, which is likely influenced by terrestrial organic run-offs.

Despite the ecological importance of Tunisian salterns, a high-resolution integrative study of their microbial and functional landscapes is still lacking. The present study aims to fill this gap by employing a multi-platform sequencing approach. We combined the depth of Illumina shotgun metagenomics with the high taxonomic resolution of full-length 16S rRNA Nanopore sequencing, supplemented by targeted 16S (V3–V4 region) and 18S (V4–V5 region) amplicon sequencing to overcome the limitations of short-read technologies. Furthermore, we moved beyond descriptive taxonomy by implementing the HUMAnN 3.0 pipeline to profile the functional metabolic pathways. This integrated strategy allowed us to: (i) characterize the taxonomic ‘dark matter’ and resolve the discrepancy between species richness and dominance in hypersaline niches; (ii) identify specific metabolic adaptations (e.g., osmolyte biosynthesis and nutrient recycling) that drive community assembly; and (iii) evaluate the impact of environmental filtering on the microbial plasticity of these extreme ecosystems.

## 2. Results and Discussion

### 2.1. Sample Characteristics

In recent years, the microbial diversity of saline ecosystems has been comprehensively explored through both culture-dependent techniques and culture-independent approaches, such as high-throughput sequencing and metagenomics [22,23]. These studies have uncovered a wealth of novel taxa and provided critical insights into their ecological roles, physiological traits, and metabolic pathways.

The physico-chemical profiles of the Thyna (Sfax) and Abbassia (Kerkennah) salterns (Table 1) reveal distinct environmental gradients that drive microbial community assembly. Although both are hypersaline, Thyna represents a more extreme habitat with a salinity of 170 g/L, compared to 105 g/L at Abbassia. This heightened osmotic pressure at Thyna is coupled with lower dissolved oxygen levels (3.5 mg/L), likely favoring obligate halophilic anaerobes. In contrast, the Abbassia site, despite its lower salinity, exhibits higher turbidity (75 NTU) and oxygenation (5.25 mg/L), suggesting a different trophic state potentially driven by higher primary productivity. The near-neutral pH and elevated temperatures (34–36 °C) at both sites confirm their status as poly-extreme habitats, where microbes must simultaneously mitigate osmotic, thermal, and oxidative stresses.

### 2.2. eDNA Extraction Protocols and Quality Control

Soil microbial communities are highly diverse but difficult to characterize due to challenges in extracting high-quality DNA from complex matrices. To improve DNA yield and purity, standard protocols of the DNeasy PowerMax Soil Kit, ZymoBIOMICS 96 MagBead DNA Kit (Zymo Research, Irvine, CA, USA), and Quick-DNA Fecal/Soil Microbe 96 MagBead Kit were optimized. Modifications included increased input material, enhanced lysis (mechanical and thermal), dialysis to remove inhibitors, and improved DNA binding and recovery steps. These adjustments resulted in higher DNA yield and sizes suitable for downstream metagenomic sequencing. Therefore, protocol modifications were introduced to optimize DNA yield, size, and integrity.

To ensure sufficient biomass for metagenomic sequencing, the initial sample matrix was doubled compared to the standard DNeasy PowerMax Soil Kit protocol. In addition, 1300 µL of lysate was loaded onto the MB spin column per round instead of the manufacturer’s recommended 600 µL, allowing larger volumes to be processed more efficiently, while a double elution step was performed to ensure maximal recovery of bound nucleic acids.

For the ZymoBIOMICS™ 96 MagBead DNA (Zymo Research, Irvine, CA, USA) as well as the Quick-DNA™ Fecal/Soil Microbe 96 MagBead kit (Zymo Research, Irvine, CA, USA), several modifications were introduced to the standard protocols. Before extraction, the soil samples were subjected to dialysis to reduce inhibitory compounds. In addition, the amount of starting material was doubled or tripled to maximize DNA recovery. The number of magnetic beads used during purification was doubled to increase nucleic acid binding capacity, and samples were mechanically disrupted for one to two hours on a Vortex Genie to improve microbial cell lysis. Heat treatment of the samples in lysis buffer was also optimized by raising the incubation temperature from 40 °C to 60 °C, which promoted more efficient cell disruption. Collectively, these modifications were introduced to improve both the purity (Table S1), yield (Table S2), and size (Figure S1) of extracted DNA, thereby ensuring the recovery of high-quality nucleic acids suitable for downstream metagenomic library preparation and sequencing.

### 2.3. Taxonomic Profiling of Archaeal Communities

A total of 16,637 reads was generated across both samples, with 16,633 reads successfully mapped, reflecting a very high mapping efficiency of over 99.9%. Specifically, the Thyna (Sfax) saltern contributed 9880 total reads (9877 mapped), while the Abbassia (Kerkennah) saltern contributed 6757 total reads (6756 mapped), providing a strong and reliable data set for downstream microbial community analysis. Both samples exhibited comparable diversity and evenness, with Simpson indices of 0.94 and Shannon indices of 4.5, indicating rich and balanced archaeal communities. At the phylum level, both samples were overwhelmingly dominated by *Euryarchaeota* (99.99%), a hallmark of hypersaline environments where high osmotic pressure filters out less specialized taxa. At the genus level, a core community of 18 shared genera (e.g., *Haloferax*, *Halorubrum*) suggests a stable functional backbone for these salterns. However, site-specific niches were clearly defined: *Halorhabdus*, exclusively found in Thyna, underscores the extreme hypersalinity (170 g/L) of this site due to its requirement for near-saturated NaCl. Conversely, the presence of *Haloterrigena* and *Haloprofundus* in only Abbassia suggests an adaptation to the specific organic matter profile and higher oxygenation (5.25 mg/L) observed in this insular system. Taxa with low abundance (<1%) were retained in the analysis to capture the ‘rare biosphere’, which may provide essential metabolic buffering to the ecosystem.

**Figure 1:**
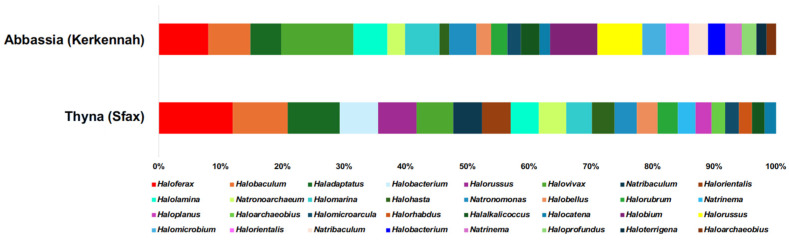
Taxonomic distribution of archaeal genera in the Abbassia (Kerkennah) and Thyna (Sfax) salterns based on Illumina MiSeq sequencing of the 16S rRNA gene (V3–V4 regions). Taxa with low abundance (<1%) were excluded.

At the genus level, 25 genera were identified across both samples (Figure 1), of which 18 were shared between the Thyna and Abbassia salterns including *Haloferax*, *Halovivax*, *Halobaculum*, *Halorussus*, *Halobacterium*, *Halomarina*, *Halolamina*, *Halorientalis*, *Natribaculum*, *Natronomonas*, *Natronoarchaeum*, *Halorubrum*, *Natrinema*, *Halobellus*, *Halalkalicoccus*, *Halocatena*, *Haloarchaeobius*, and *Halomicroarcula*, presenting relative abundances ranging from 1.01% to 7.45% in the Thyna saltern, and 1.01% to 7.7% in the Abbassia saltern, indicating that they form the dominant fraction of the archaeal communities in both samples.

These genera belong to well-characterized families within the orders *Halobacteriales*, *Haloferacales*, and *Natrialbales*, which collectively encompass the majority of described haloarchaeal diversity in hypersaline systems [24,25,26]. Ecologically, genera such as *Haloferax*, *Halorubrum*, *Halolamina*, and *Natronomonas* are abundant in hypersaline soils and sediments, often correlating with salinity gradients, and contributing substantially to community structure. Other taxa, including *Halovivax* and *Halobaculum*, are also often documented to be present in saline ecosystems, though typically at lower abundance or in specific niches such as soil crusts or salt crust layers [24,26].

Certain unique haloarchaeal genera were identified at specific sites; for instance, *Halohasta*, *Haloplanus,* and *Halorhabdus* were only found in Thyna, whereas *Halobium*, *Halomicrobium*, *Haloprofundus,* and *Haloterrigena* were only found in Abbassia. These genera were present at low relative abundances (Figure 1). *Halohasta* species are extremophilic archaea frequently identified in salt-rich environments and contribute to community resilience through their capacity to withstand high ionic strength, often co-occurring with other haloarchaea across salterns and saline soils, suggesting niche specialization along salinity gradients [26]. *Haloplanus* species are reported to be extreme halophiles characterized by mesophilic growth and pigmentation. Genomic analyses indicate their capabilities for broad heterotrophic metabolism and stress tolerance, traits that support survival in fluctuating salinity and nutrient conditions typical of solar salterns [27]. Members of the genus *Halorhabdus* are notable for their extremely high salinity optimum (up to ~27% NaCl) and restricted substrate range, reflecting a specialized ecological strategy for growth under saturated salt conditions. Their morphology and metabolic preferences further underscore their adaptation to extreme halophily [28]. *Halobium* comprises extremely halophilic archaea, which are capable of growing at the high concentrations of NaCl typical of solar salterns. Their major polar lipids, such as phosphatidylglycerol and sulfated glycolipids, indicate adaptations to osmotic stress in their membranes [29]. The genus *Halomicrobium* belongs to the *Haloarculaceae* family and is commonly found in hypersaline environments, which suggests a broad range of metabolic processes that allow for survival under high salt conditions [30]. *Haloprofundus* comprises highly salt-tolerant haloarchaea that have been isolated from saline soils and sediments [31]. These organisms have phenotypic characteristics that shows adaptation to extreme salt and nutrient-limited conditions, while *Haloterrigena* comprises pleomorphic, extremely halophilic archaea that grow optimally at high NaCl concentrations and moderate temperatures, with genomic features that support survival in salt-rich habitats [32].

### 2.4. Taxonomic Profiling of Eukaryotic Communities

The two saline soil samples from the Thyna (Sfax) and Abbassia (Kerkennah) salterns generated over one million 18S reads each, with 618,889 (48.5%) and 581,854 (50.8%) reads mapped to OTUs, respectively. Classification of these reads revealed that only 28% of Thyna’s reads (≈357,776 reads) and 51% of Abbassia’s reads (≈584,918 reads) could be assigned to known taxa, indicating a higher proportion of uncharacterized eukaryotes in the Thyna sample compared to the Abbassia sample. The high percentage of unassigned reads (up to 72% in Thyna) represents a significant finding. This observation is due to the lack of representative sequences for extreme hypersaline eukaryotic lineages in public databases (SILVA/PR2). This can suggest that hypersaline soils in Tunisia are reservoirs for previously unreported fungal diversity. To our knowledge, no comprehensive metagenomic study has yet focused on the eukaryotic communities inhabiting these Tunisian hypersaline soils.

Alpha diversity analysis confirmed that both communities are highly diverse and evenly distributed, with Simpson indices of 0.95 (Thyna) and 0.96 (Abbassia), and Shannon indices of 5.3 and 6.0, respectively. These results suggest that saline soils harbor complex microbial communities, with Thyna containing a greater fraction of potentially novel halotolerant fungi or other extremophilic eukaryotes, while Abbassia exhibits slightly higher overall diversity, reflecting subtle ecological differences between the sites. For example, fungi are known to play a crucial role in breaking down complex polymers (such as lignin or cellulose) [33]. These polymers may be more available in the Thyna sediments due to the saltern’s proximity to the mainland and potential terrigenous exposure.

**Figure 2:**
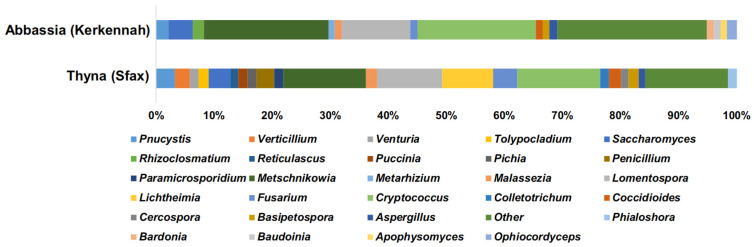
Bar plots representing the relative abundance of fungal communities at the genus level in the Abbassia and Thyna salterns. Taxonomic profiles were generated using full-length 18S rRNA (V4–V5) region Illumina sequencing. Genera with a relative abundance of less than 1% were filtered for clarity.

At the phylum level, both salterns were dominated by *Ascomycota*, comprising 69% and 84% in Thyna and Abbassia, respectively, highlighting the prevalence of this major fungal lineage in saline environments. *Basidiomycota* were more abundant in the Thyna (19%) saltern than in the Abbassia (7.9%) saltern, while *Mucoromycota* were also higher in the Thyna (8.7%) saltern compared to the Abbassia (1.8%) saltern. Minor fractions included *Cryptomycota* (1% in Thyna) and other unclassified fungi (~1–1.5% in both soils) (Figure 2). These differences suggest that the Thyna saltern harbors a more phylogenetically diverse fungal community at the phylum level, whereas the Abbassia saltern is more dominated by *Ascomycota*.

At the genus level, the soil saltern samples collected from Abbassia and Thyna revealed a diverse community of microscopic fungi dominated by both yeasts and filamentous taxa. In Thyna, the most abundant genera included *Metschnikowia* (14%), *Cryptococcus* (14%), *Lomentospora* (11%), and *Lichtheimia* (8.6%), whereas Abbassia was dominated by *Metschnikowia* (20%) and *Cryptococcus* (19%), indicating a consistent yeast-rich component across both saline soils. The presence of genera such as *Lomentospora* and *Lichtheimia*, often associated with nutrient-rich or anthropized environments, further differentiates the ecological profile of Thyna from the more isolated Abbassia site.

Filamentous genera such as *Fusarium*, *Verticillium*, *Penicillium*, *Aspergillus*, *Colletotrichum*, *Tolypocladium*, *Reticulascus*, *Ophiocordyceps*, *Phialophora*, *Lomentospora*, *Lichtheimia*, *Apophysomyces*, *Baudoinia*, and *Puccinia* were detected at lower relative abundances, reflecting a heterogeneous assemblage of stress-tolerant fungi. Notably, several OTUs could only be assigned at higher taxonomic levels (e.g., *Piptocephalidaceae*, *Cephalotrichiella*) or represented novel and poorly characterized fungal lineages, suggesting the presence of previously unreported diversity in saline soils. These results indicate that saline soils, such as those at Abbassia and Thyna, harbor a specialized fungal community dominated by osmotolerant yeasts and filamentous fungi, likely adapted to high salinity and low water activity. The consistent presence of genera such as *Metschnikowia* and *Cryptococcus*, known for their halotolerance and osmoprotectant production, underscores their ecological relevance in extreme soil environments. The eukaryotic profiling of the Thyna and Abbassia salterns reveals a remarkably high proportion of unassigned 18S rRNA sequences (up to 72% in Thyna), a phenomenon frequently described as ‘taxonomic dark matter’ in extreme environments [34]. This significant gap in public databases, such as SILVA or PR2, suggests that Tunisian solar salterns harbor a vast, yet-to-be-characterized reservoir of extremophilic eukaryotes, specifically adapted to the unique geochemical constraints of the Mediterranean basin [35]. The dominance of *Ascomycota* (69–84%) across both sites is consistent with their known plasticity and ability to withstand high osmotic pressure through the ‘High-Osmolarity Glycerol’ (HOG) signaling pathway [36]. Interestingly, the prevalence of yeast genera such as *Metschnikowia* and *Cryptococcus* suggests a specialized ecological niche. These taxa are recognized for their ability to accumulate polyols and synthesize thick polysaccharide capsules, which serve as desiccation barriers in hypersaline soils [37]. The higher abundance of *Lomentospora* and *Lichtheimia* in Thyna compared to Abbassia may reflect the influence of terrigenous inputs and anthropogenic proximity. These filamentous fungi are often saprotrophic, playing a pivotal role in the turnover of complex organic matter (lignocellulose) that reaches the salterns from the mainland [38]. This functional specialization, combined with the presence of rare phyla such as *Cryptomycota*, underscores that these salterns are not merely extreme ‘stress zones’, but complex ecosystems where fungal communities ensure nutrient cycling under near-saturation salinity [39].

These specialized fungal communities not only provide a baseline for exploring novel halophilic or halotolerant fungi in extreme environments but also serve as a foundation for exploring novel enzymes and secondary metabolites with potential biotechnological applications.

### 2.5. Taxonomic Profiling of Bacterial Communities

To characterize the bacterial community structure of the two samples, we performed full-length 16S rRNA gene sequencing (covering the V1–V9 regions). High-resolution data were obtained using the ONT PromethION 2 Solo platform, complemented by shotgun metagenomic sequencing performed with both Illumina and Nanopore technologies.

Shotgun metagenomic sequencing using Illumina technology generated 8,004,385 reads for Abbassia and 15,498,770 reads for Thyna. In contrast, Nanopore-based shotgun sequencing yielded 534,369 reads for Abbassia and only 104 reads for Thyna. For 16S rRNA sequencing using Nanopore technology, 2,139,081 reads were obtained from Abbassia, while 3,703,450 reads were generated from Thyna.

Rarefaction analysis was used to assess sequencing depth and species richness across samples by subsampling to a uniform depth and plotting observed taxa as a function of sequencing technology (Figure S2). Results revealed marked differences in species richness and sequencing depth across the three sequencing approaches. Shotgun Illumina samples (Thyna and Abbassia) exhibited the highest richness, plateauing at ~1500 and ~1300 species, respectively, indicating sufficient sequencing depth and highly diverse communities. Nanopore samples showed lower richness: Thyna 16S (~700), Shotgun Nanopore Abbassia (~500), Abbassia 16S (~50), and Shotgun Nanopore Thyna near zero, reflecting reduced diversity and possible technical limitations. Overall, Illumina sequencing captured substantially greater microbial diversity, while Nanopore data sets displayed lower richness and higher variability. Based on these rarefaction results, the Abbassia 16S and Shotgun Nanopore Thyna data sets were excluded from subsequent analyses to prevent statistical artifacts and ensure that the ecological interpretations were based only on robust, high-depth data sets.

**Figure 3:**
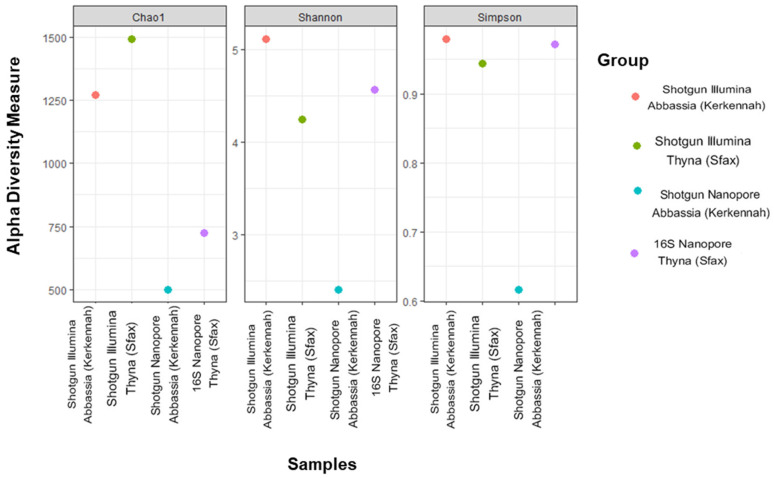
Alpha diversity of microbial communities for the Thyna and Abbassia samples derived from different sequencing approaches. Diversity was assessed using Chao1 (species richness), Shannon (richness and evenness), and Simpson (evenness/dominance) indices.

Alpha diversity was evaluated across the different selected sequencing approaches for the Thyna and Abbassia samples using Chao1, Shannon, and Simpson indices after normalization to account for sequencing depth (Figure 3).

Results revealed marked differences in microbial community structure across samples and sequencing platforms. Species richness (Chao1) was highest in Shotgun_Illumina_Thyna (~1500) and Shotgun_Illumina_Abbassia (~1250), but moderate in the Thyna 16S Nanopore (~700) and Shotgun_Nanopore_ Abbassia (~500) sequence data. Shannon diversity, reflecting both richness and evenness, followed a similar trend: Shotgun_Illumina_Abbassia (~5.1), Thyna 16S Nanopore (~4.5), Shotgun_Illumina_Thyna (~4.2), with lower values for Nanopore Abbassia (~2.4), and minimal diversity in other Nanopore samples. The Simpson index, emphasizing dominance, indicated highly even communities in Illumina samples and Thyna 16S Nanopore (~1), moderate evenness in Shotgun_Nanopore_Abbassia (~0.6), low evenness in Abbassia 16S (~0.08), and near-zero evenness in Shotgun_Nanopore_Thyna. Overall, Illumina-based shotgun sequencing captured the most diverse and balanced communities, whereas Nanopore data sets exhibited reduced richness, uneven distributions, and higher dominance effects, highlighting both biological variation and platform-specific biases.

**Figure 4:**
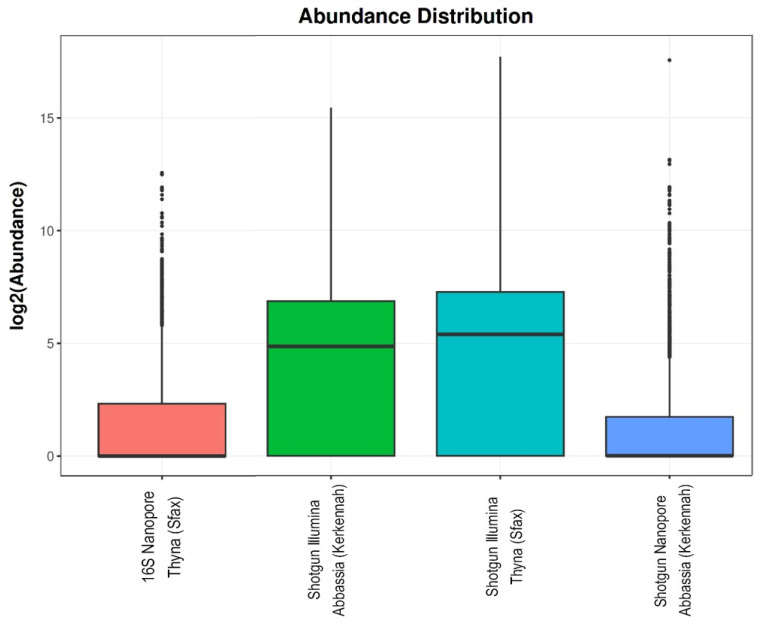
Abundance distribution of microbial taxa across different sequence data sets. Boxplots represent log_2_(x + 1)-transformed counts, summarizing the median, interquartile range, and variability of abundances for each data set. Outliers indicate taxa with exceptionally high or low abundance. This visualization highlights differences in microbial community structure between sequencing methods (16S rRNA vs. shotgun) and sampling sites (Thyna vs. Abbassia).

The abundance distribution of microbial taxa across the different data sets was visualized using a boxplot of log-transformed values. Raw counts were transformed with log_2_(x + 1) to mitigate the influence of highly abundant taxa and stabilize variance, enabling clearer comparison between data sets. Each box in the plot represents a single sample and summarizes the range, median, and variability of abundance values across all detected taxa or operational taxonomic units. This approach provides a concise overview of community composition and highlights differences in microbial feature distributions among the sample data sets (Figure 4).

The boxplots reveal differences in community structure between sequencing approaches and sampling sites. Shotgun Illumina data sets from both Thyna and Abbassia exhibit higher median abundance and wider variability compared with Nanopore data sets, reflecting broader coverage of microbial features. In contrast, 16S rRNA Nanopore data sets show lower median abundances and a greater proportion of low-abundance taxa, consistent with the targeted nature of amplicon sequencing. These patterns illustrate how the sequencing method and site-specific microbial composition influence the distribution of taxonomic abundances and highlight the presence of both dominant and rare taxa across hypersaline sediments.

**Figure 5:**
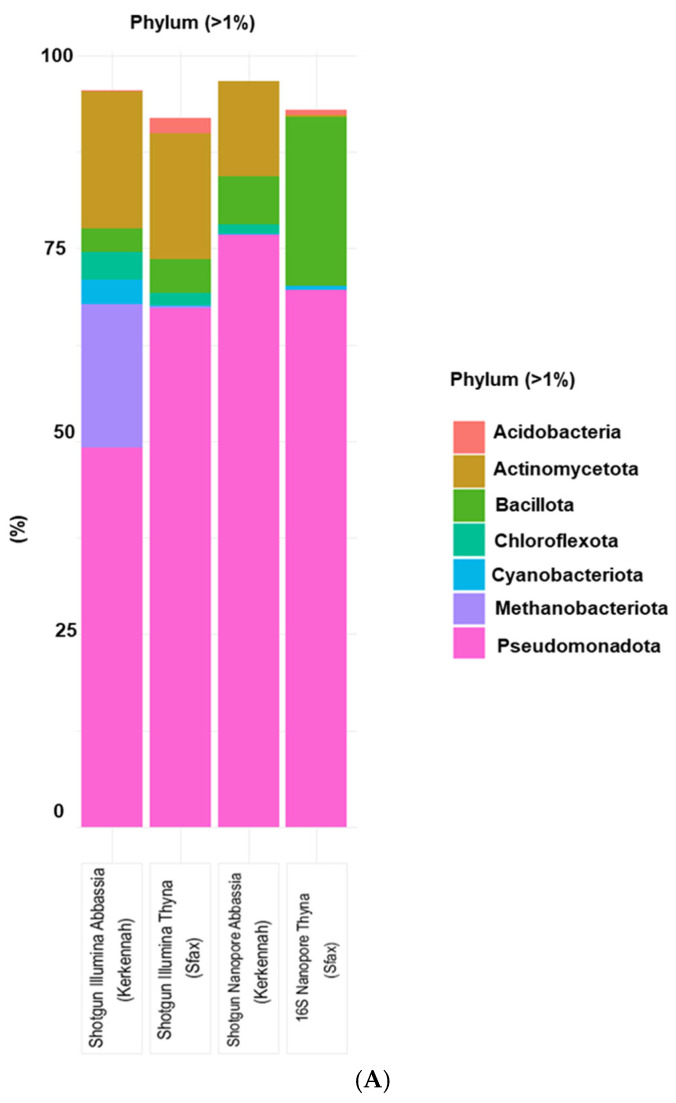

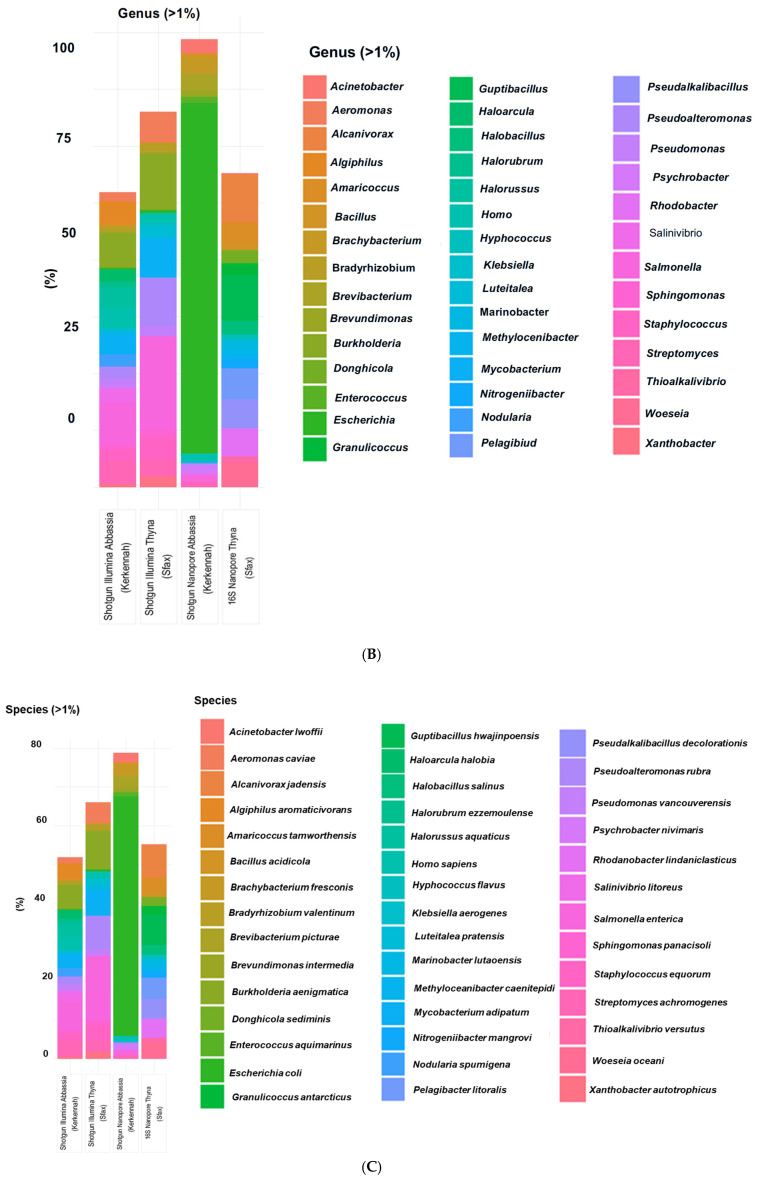
Phylum (**A**), genus (**B**), and species-level (**C**) distribution of microbial communities in the Abbassia and Thyna salterns. Comparison of relative abundances (%) across 16S rRNA Nanopore amplicons and shotgun metagenomics data sets generated using Illumina and Nanopore sequencing platforms. Only taxa with relative abundance > 1% are shown.

The microbial community composition across the two sites with relative abundance >1% is presented in Figure 5. At the phylum level, microbial communities exhibit a broadly consistent structure across all samples, with the *Pseudomonadota* group being dominant. Its relative abundance is approximately 50% in Shotgun_Illumina_Kerkennah, 67% in Shotgun_Illumina_Thyna, 78% in Shotgun_Nanopore_Abbassia, and 69–70% in Thyna_16S_Nanopore. The predominance of *Pseudomonadota* is widely reported in metagenomic studies in saline ecosystems, where this phylum is considered a core component due to its metabolic versatility and adaptability to fluctuating salinity conditions [40,41]. The second most abundant phylum is *Actinomycetota*, which contributes approximately 18–20% in Shotgun_Illumina_Abbassia, 15–17% in Shotgun_Illumina_Thyna, 12–15% in Shotgun_Nanopore_Abbassia, and 20–22% in Thyna_16S_Nanopore. The significant presence of *Actinomycetota* is noteworthy, as members of this phylum are renowned for their ability to produce secondary metabolites and exoenzymes that degrade complex organic matter, a crucial function in nutrient-limited hypersaline sediments [42]. Similarly, the *Bacillota* phylum accounts for approximately 18%, 20%, 10%, and 22% in the same data sets, respectively. Members of these phyla are commonly reported in hypersaline and fluctuating environments studies due to their ability to form endospores and survive osmotic stress [43]. Minor phyla, including *Bacteroidota* and *Cyanobacteriota*, represent less than 5–8% of the total community.

At the genus level, clear differences in dominance and diversity are observed within each site when comparing sequencing technologies. At the Abbassia site, the shotgun Illumina-based sequence data reveals a relatively even and diverse community, dominated by *Pseudomonas* (~14–16%), followed by the *Brevundimonas/Bradyrhizobium* group (~8–10%) and *Acinetobacter* (~6–8%), with additional genera such as *Bacillus* and *Sphingomonas,* each contributing less than 5%.

This pattern is consistent with earlier research showing that these bacterial groups have been widely reported in natural saline ecosystems [44]. *Pseudomonas* is ubiquitous in saline systems due to its metabolic versatility and osmotic stress tolerance [45], while *Bradyrhizobium* and *Brevundimonas* are involved in nutrient cycling in oligotrophic and saline conditions [46], while *Acinetobacter* is frequently detected in brackish and stressed environments [47]. It is worth noting that the shotgun Nanopore data set is dominated by a single genus, likely *Escherichia*/*Shigella* (~65–70%), with all other genera, including *Pseudomonas* and *Bacillus*, being below 5%. This discrepancy suggests either strong local environmental selection or a methodological bias, as long-read shotgun approaches can overrepresent highly abundant taxons.

At the Thyna site, the shotgun Illumina data set shows partial dominance by *Pseudomonas* (~25–28%), followed by *Burkholderia*/*Paraburkholderia* (~12–15%) and *Bacillus* (~10–12%), with other genera contributing between 3% and 8%. *Burkholderia*/*Paraburkholderia* are commonly enriched in saline and contaminated environments due to their roles in nitrogen cycling and degradation of complex compounds [48], while *Bacillus* is well known for its resilience in fluctuating salinity due to endospore formation [49,50]. In comparison, the 16S Nanopore data set (Thyna_16S_Nanopore) displays a more even distribution, with *Pseudomonas* (~12–15%), *Bacillus* (~10–12%), and *Acinetobacter* (~8–0%), along with several genera in the 5–10% range.

Overall, comparing genus-level composition within each site across sequencing technologies reveals that Illumina-based approaches tend to capture greater diversity and evenness, while Nanopore data, particularly shotgun, may emphasize dominant taxa. Despite these differences, recurrent detection of key genera such as *Pseudomonas*, *Bacillus*, and *Acinetobacter* across both methods supports their central ecological role in saline environments.

At the species level, the observed patterns closely mirror those at the genus level but provide finer ecological resolution, revealing important differences in community structure across sites that are consistent with dynamics typically reported in saline ecosystems. At the species level, shotgun metagenomics uniquely allowed for the simultaneous detection of both bacterial and archaeal members, such as *Halorubrum* spp. (5%) in Abbassia, providing a more integrated view of the prokaryotic community than 16S rRNA sequencing alone. The observed patterns closely mirror those at the genus level but provide finer ecological resolution. In Abbassia (Kerkennah), the community is relatively diverse and well-balanced, with several halotolerant and metabolically versatile species distributed across different taxa. The dominant species include *Pseudomonas aeruginosa* (10%), *Pseudomonas fluorescens* (8%), *Bacillus subtilis* (10%), *Sphingomonas paucimobilis* (6%), and members of the *Burkholderia cepacia* group (5%).

In contrast, Thyna (Sfax) shows a more selective and uneven structure, with stronger dominance of a few bacterial groups. The most abundant species include *P. aeruginosa* (25%), *B. subtilis* (15%), and *Pseudomonas putida*, while other taxa appear at lower abundances. Halotolerant organisms such as *Halomonas elongata* are present but less prominent. Additional opportunistic species such as *Staphylococcus aureus* and *Streptomyces* spp. occur in low proportions. This pattern indicates a stronger ecological filtering in Thyna, leading to reduced diversity and dominance of a few adaptable species.

### 2.6. Functional Profiling of Microbial Communities

**Figure 6:**
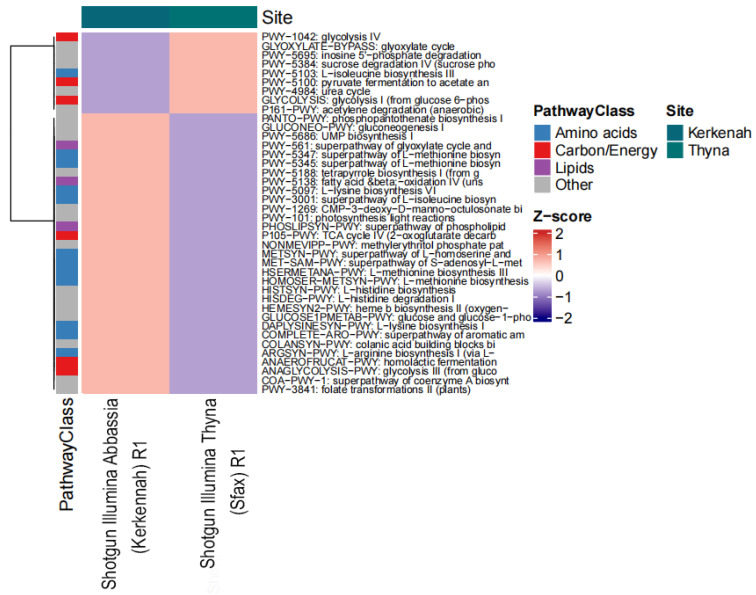
Differential abundance of the top 40 functional pathways (Z-Score) across the Abbassia and Thyna microbial communities.

Functional annotation of Illumina-derived data sets was performed using the HUMAnN 3.0 pipeline in conjunction with the MetaCyc database (v29.1), allowing for the reconstruction and quantification of gene family abundances (Table S3) and metabolic pathway coverage across samples (Tables S4 and S5). The results revealed that both sites shared a core set of central metabolic pathways, including glycolysis (Glycolysis, PWY-5484, and PWY-1042), the pentose phosphate pathway (Nonoxipent-PWY), and components of the TriCarboxylic Acid (TCA) cycle, indicating conserved basal metabolic functions across hypersaline environments. Nevertheless, there were clear differences in the number and location of pathways, which is an indication of different metabolic strategies (S_abundance). The differential abundance profiles of the top 40 functional pathways across the Abbassia and Thyna sites are shown in Figure 6.

**Figure 7:**
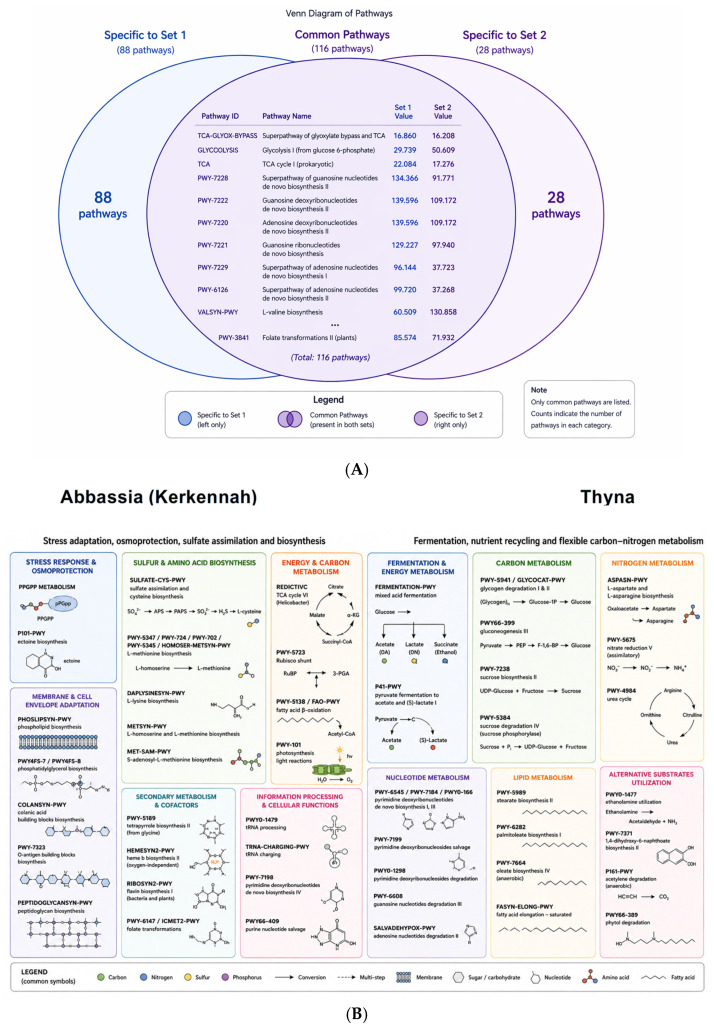
Comparison of metabolic pathways at Abbassia and Thyna sites. (**A**) Venn diagram illustrating the overlap between metabolic pathways identified in the Abbassia and Thyna sites, highlighting both shared and site-specific functions. (**B**) Schematic representation of pathways unique to each site, providing a detailed view of site-specific metabolic capabilities and functional differentiation between the two environments.

The Abbassia microbiome was characterized by a significant abundance of anabolic and biosynthetic pathways (Figure 7). Notably, multiple amino acid biosynthesis pathways were present in high abundance, including those for arginine (ARGSYN-PWY and PWY-5154), lysine (DAPLYSINESYN-PWY and PWY-5097), histidine (HISTSYN-PWY), tryptophan (TRPSYN-PWY and PWY-6629), and branched-chain amino acids (BRANCHED-CHAIN-AA-SYN-PWY, VAL-SYN-PWY, and ILEUSYN-PWY). In saline ecosystems, a high abundance of amino acid biosynthesis pathways is a key adaptation strategy enabling microbial communities to thrive under nutrient-limited or fluctuating conditions [51]. Additionally, elevated levels of nucleotide biosynthesis pathways were observed, particularly those involved in de novo purine and pyrimidine synthesis (PWY-7220, PWY-7221, PWY-7222, and PWY-6125/PWY-6126), as well as cofactor and vitamin metabolism, including folate transformations (PWY-3841) and coenzyme A biosynthesis (COA-PWY and PANTOSYN-PWY).

Elevated investment in nucleotide synthesis is typically linked to increased cellular replication and turnover, reflecting dynamic microbial growth under favorable or competitive conditions [52]. The enrichment of pathways related to cofactor and vitamin metabolism, such as folate conversions and coenzyme, emphasizes the significance of metabolic adaptability and redox equilibrium within this ecosystem. These cofactors are essential for a wide range of enzymatic reactions, suggesting that a robust biochemical network is maintained by the microbiome to support diverse metabolic functions [53]. Pathways involved in heme biosynthesis (HEME-BIOSYNTHESIS-II and HEMESYN2-PWY) and lipid metabolism, including fatty acid *β*-oxidation (FAO-PWY and PWY-5136), were also significantly enriched.

Notably, the Abbassia microbiome showed a distinctive enrichment of stress-adaptive and protective pathways, such as ectoine biosynthesis (P101-PWY) and ppGpp metabolism (PPGPPMET-PWY). Ectoine is a well-known compatible solute that stabilizes proteins and membranes under high salinity. This indicates that the microbial communities in Abbassia actively mitigate osmotic stress [54]. Similarly, ppGpp metabolism reflects the activation of the stringent response, a global regulatory mechanism that enables bacteria to survive under conditions of limited nutrients and environmental stress by reallocating cellular resources [55]. Secondly, the enrichment of multiple membrane and cell envelope biosynthesis pathways suggests structural adaptation of the cell envelope. Examples of these pathways include phospholipid biosynthesis, peptidoglycan formation, colanic acid production, and O-antigen biosynthesis. In saline conditions, it is critical that membrane integrity and fluidity are maintained, enhancing resistance against ionic stress, and dehydration is likely to be contributed to by these pathways [56]. Membrane remodeling and energy production under fluctuating environmental conditions are further supported by the presence of fatty acid metabolism (*β*-oxidation and cis-vaccenate biosynthesis) [57]. Another key aspect is the overrepresentation of amino acid and sulfur metabolism, including SULFATE-CYS-PWY and multiple methionine biosynthesis pathways. This indicates a strong reliance on inorganic sulfate assimilation to sustain the production of sulfur-containing amino acids, which are essential for protein synthesis and redox homeostasis. In saline ecosystems where organic sulfur is often limited, this metabolic flexibility provides a significant ecological advantage [58].

The Abbassia communities also show enrichment in central carbon metabolism and energy pathways, such as variations in the TCA cycle, the Rubisco shunt, and *β*-oxidation. These pathways suggest metabolic versatility, allowing microorganisms to exploit diverse carbon sources and maintain energy production under stress conditions [59]. The detection of photosynthesis-related pathways and tetrapyrrole/heme biosynthesis further indicates the presence of phototrophic or photo-assisted metabolisms, which can be advantageous in shallow, high-salinity coastal environments. Finally, the abundance of pathways related to nucleotide biosynthesis, tRNA charging, and cofactor production (e.g., NAD salvage, flavin biosynthesis, folate transformations) reflects a high level of cellular activity and turnover, suggesting that, despite harsh conditions, Abbassia supports metabolically active and dynamically adapting microbial populations.

With regard to taxonomic composition, the community is dominated by a *Pseudomonadota*/*Gammaproteobacteria*-like lineage (60%), indicating the prevalence of metabolically versatile marine heterotrophs and opportunistic degraders that can rapidly respond to organic substrates [60]. A substantial secondary contribution is observed from *Actinomycetota*-related groups (25%), which are commonly associated with the decomposition of organic matter and the production of secondary metabolites, suggesting their active participation in carbon cycling processes [61]. Additionally, a distinct archaeal fraction, represented by *Halobacteria* (15%), reflects adaptation to saline or hypersaline environmental conditions, which are typical of marine-influenced ecosystems [24]. Minor contributions from taxa such as *Cyanobacteria* indicate the presence of phototrophic or background environmental microorganisms, though these do not dominate the community structure [62]. The observed taxonomic structure is consistent with the functional pathway profile, which is enriched in pathways related to energy metabolism, biosynthetic processes, and nucleotide turnover.

By contrast, the Thyna microbiome showed a functional profile that was characterized by pathways associated with catabolism and the generation of energy (Figure 7). Fermentation pathways were particularly prominent, including mixed acid fermentation (FERMENTATION-PWY), pyruvate fermentation to acetate and lactate (P41-PWY and PWY-5100), and alternative fermentative routes, such as *iso*-butanol production (PWY-7111). There was also an increased abundance of pathways involved in carbohydrate metabolism, including glycolysis (GLYCOLYSIS), gluconeogenesis (PWY66-399), sucrose degradation (PWY-5384), and glycogen turnover (GLYCOCAT-PWY, PWY-5941). A striking feature of the Thyna functional profile was the strong enrichment of the glyoxylate cycle. This is also known as the glyoxylate bypass (GLYOXYLATE-BYPASS; PWY-5690). This suggests an adaptive strategy. This strategy is for the conservation and utilization of simple carbon substrates. These are used under nutrient-limited or stress conditions. Additionally, Thyna samples exhibited a higher representation of nucleotide degradation and salvage pathways, including purine and pyrimidine turnover (PWY0-1296, PWY-6608, PWY-7199, and SALVADEHYPOX-PWY), indicating a reliance on recycling mechanisms rather than de novo synthesis. Several pathways related to nitrogen metabolism, such as assimilatory nitrate reduction (PWY-5675), were also detected predominantly in Thyna, consistent with the presence of nitrifying taxa identified in the taxonomic analysis.

Site-specific enrichment was characterized by the strong presence of fermentation-based energy pathways, including FERMENTATION-PWY (mixed acid fermentation), P41-PWY (pyruvate fermentation to acetate and (S)-lactate I), and PWY-7111 (pyruvate fermentation to *iso*-butanol). The presence of these pathways indicates that microbial communities depend significantly on anaerobic or microaerophilic metabolism, a process that involves the phosphorylation of substrates to produce energy [63]. Indeed, carbon storage and mobilization are also significant processes, as demonstrated by GLYCOCAT-PWY (glycogen degradation I) and PWY-5941 (glycogen degradation II), coupled with PWY66-399 (gluconeogenesis III). A dynamic carbon buffering system is reflected in these pathways, allowing microorganisms to alternate between storage and utilization depending on environmental conditions. The presence of PWY-7238 (sucrose biosynthesis II) and PWY-5384 (sucrose degradation IV) further suggests the active turnover of plant-derived carbohydrates, which is consistent with the coastal input of organic matter [64].

Metabolism of nitrogen and nucleotides is also highly represented. The ASPASN-PWY pathway (superpathway of L-aspartate and L-asparagine biosynthesis) supports amino acid synthesis under variable nutrient conditions. Meanwhile, PWY-5675 (nitrate reduction V—assimilatory) demonstrates the ability of community members to utilize nitrate as a source of nitrogen [65]. Meanwhile, multiple nucleotide recycling and synthesis pathways, such as PWY-6545 (pyrimidine deoxyribonucleotides de novo biosynthesis III), PWY-7184 (pyrimidine deoxyribonucleotides de novo biosynthesis I), PWY-0166 (superpathway of pyrimidine deoxyribonucleotides de novo biosynthesis), PWY-7199 (pyrimidine deoxyribonucleosides salvage), and PWY-66389 (phytol degradation), indicate strong nucleotide turnover and salvage metabolism, which is typical of environments where microbial growth fluctuates with resource availability.

Another important component is lipid metabolism, with pathways such as PWY-5989 (stearate biosynthesis II), FASYN-ELONG-PWY (fatty acid elongation—saturated), PWY-6282 (palmitoleate biosynthesis I), and PWY-7664 (oleate biosynthesis IV—anaerobic). Together, these pathways suggest the capacity for membrane remodeling, enabling adaptation to changing salinity and redox conditions. However, they lack the strong osmoprotective specialization observed in Abbassia [66].

Thyna microbial communities exhibit nitrogen recycling and central metabolic flexibility. PWY-7371 (1,4-dihydroxy-6-naphthoate biosynthesis II), PWY-6608 (guanosine nucleotide degradation III), SALVADEHYPOX-PWY (adenosine nucleotide degradation II), and PWY-6282 (phytol degradation) all contribute to the efficient reuse of organic compounds. The presence of PWY-4984 (the urea cycle) also supports the detoxification and recycling of nitrogen, which is likely to be important in fluctuating organic loads [67]. Finally, pathways such as PWY0-1477 (ethanolamine utilization) and PWY-7199 (pyrimidine deoxyribonucleoside salvage) demonstrate the capacity to utilize various anthropogenic or degradation-derived substrates, emphasizing the concept of a heterotrophic, opportunistic microbial community [68].

The microbial community in Thyna is dominated by *Pseudomonadota* (55%), followed by *Actinomycetota* (25%) and methanogenic *Methanobacteriota* (20%), reflecting a metabolically stratified ecosystem combining aerobic, facultative, and strictly anaerobic processes. The prevalence of *Pseudomonadota*-associated genera such as *Pseudomonas* and *Acinetobacter* is consistent with their well-documented metabolic versatility and ability to thrive under fluctuating redox conditions, supporting pathways involved in glycolysis, gluconeogenesis, and fermentation (e.g., pyruvate-to-acetate and mixed-acid fermentation). These taxa are widely recognized for their roles in degrading organic matter and adapting to energy metabolism in marine and coastal environments [69,70].

The substantial contribution of *Actinomycetota* further supports this interpretation, as members of this phylum, including *Streptomyces*, are key decomposers of complex organic substrates and producers of secondary metabolites, contributing to carbon turnover and nutrient recycling [71].

The relatively high abundance of methanogenic archaea (*Methanobacteriota*) observed correlates with the presence of the methanogenesis and hydrogenotrophic pathways. These pathways suggest the existence of anoxic microenvironments where terminal carbon mineralization occurs via methane production. In fact, methanogens such as *Methanobacterium* are known to utilize H_2_ and CO_2_ as substrates, playing a central role in anaerobic carbon cycling and linking fermentative bacterial processes to final energy-yielding steps [72,73]. The prevalence of methanogenic *Methanobacteriota* and fermentation pathways in Thyna is consistent with the lower dissolved oxygen levels (3.5 mg/L) recorded at this site, favoring the development of anoxic micro-niches. The coexistence of fermentative bacteria and methanogenic archaea suggests a syntrophic relationship, in which bacterial degradation products (e.g., acetate, H_2_, and CO_2_) fuel archaeal methanogenesis, a well-established mechanism in anoxic sediments and wastewater-influenced environments [74].

Taken together, Abbassia is dominated by biosynthetic and anabolic processes, indicative of a growth-oriented and metabolically self-sufficient community. In contrast, the Thyna microbial community exhibits a metabolism centered on energy extraction, fermentation, and nutrient recycling, reflecting a more dynamic and environmentally responsive ecosystem. These findings provide strong evidence that environmental conditions shape not only taxonomic composition but also the functional potential of microbial communities in hypersaline sediments.

## 3. Materials and Methods

### 3.1. Site Description and Sample Collection

Sediment samples were collected from two industrial solar salterns located in the Sfax Governorate, Tunisia: Abbassia (Kerkennah Island; 34.6833° N, 11.1167° E), which is characterized by high evaporation rates and limited anthropogenic impact, whereas Thyna (Sfax; GPS coordinates: 34.7200° N, 10.7600° E) is subject to seasonal fluctuations and urban proximity. Both sites are engineered systems designed for industrial salt production through a series of evaporation pans. Sampling was conducted in July, during the peak of the dry season, to capture the microbial communities under maximum hypersaline and thermal stress. At each site, surface sediments (top 5 cm) were collected from the bottom of the saline ponds using sterile spatulas. To minimize the impact of micro-scale sediment heterogeneity and capture a representative average of each site’s microbial signature, a pooled composite sampling strategy was employed for the metagenomic analysis. The collected sediments were immediately preserved in DNA/RNA Shield (Zymo Research, Irvine, CA, USA) to stabilize nucleic acids, transported to the laboratory on ice within 4 h, and immediately frozen at −80 °C until DNA extraction. Environmental parameters of the overlying brine and the sediment interface were recorded (Table 1). The physico-chemical parameters measured at the time of sampling served as environmental metadata for subsequent correlation with microbial diversity and functional profiles.

The pH was determined using a METTLER TOLEDO SevenEasy™ S20 pH meter. Salinity was measured gravimetrically by drying 10 mL of the interstitial brine at 105 °C until reaching a constant weight. Water temperature was recorded in situ using an AcuRite 8” Analog Thermometer (AcuRite/Chaney Instrument Co., Lake Geneva, WI, USA).

### 3.2. Environmental DNA Extraction, Quality Assessment, and Quantification

To optimize the recovery of high-quality metagenomic DNA suitable for both short-read (Illumina) and long-read (Nanopore) sequencing, an initial optimization phase was conducted comparing three extraction methods: the DNeasy PowerMax Soil Kit (Qiagen, Hilden, Germany), the ZymoBIOMICS™ 96 MagBead DNA Kit (Zymo Research, Irvine, CA, USA), and the Quick-DNA Fecal/Soil Microbe 96 MagBead Kit (Zymo Research, Irvine, CA, USA). Based on superior performance in terms of DNA yield and purity, the ZymoBIOMICS™ 96 MagBead DNA Kit (Zymo Research, Irvine, CA, USA) was selected as the standardized method for all samples analyzed in this study. For each site, a composite sediment sample (formed by physically mixing multiple cores) was used for environmental DNA (eDNA) extraction. The manufacturer’s protocol was followed with minor modifications, including additional washing steps to account for the high salinity of the matrix and ensure the removal of potential PCR inhibitors. This standardized approach was applied consistently across all sites to eliminate potential kit-specific extraction biases. For each kit, extraction blanks (reagents only, no sediment material) were processed for every batch of samples to monitor for any ‘kitome’ or reagent-derived DNA. No additional chemical cleaning steps were required before library preparation, as the optimized extraction successfully removed inhibitory humic substances and salts.

The concentration and purity of the extracted eDNA were initially assessed using an Implen™ Nanophotometer™ N60/N50 (Implen GmbH, Munich, Germany). The obtained A_260_/A_280_ ratios ranged between 1.8 and 2.0, while A_260_/A_230_ ratios were above 1.5, confirming high-quality nucleic acids.

Precise quantification was subsequently performed using a Quantus™ Fluorometer (Promega, Madison, WI, USA) with the QuantiFluor^®^ dsDNA System.

To ensure the structural integrity required for long-read metagenomics, DNA size distribution and degradation levels were rigorously evaluated using the 5300 Fragment Analyzer system (Agilent Technologies, Lawrence, KS, USA). The Agilent DNF-464-FR HS LRG Fragment 50 kb and DNF-492-FR Large Fragment kits were utilized to confirm the presence of high-molecular-weight DNA fragments.

### 3.3. Illumina Library Preparation and Bioinformatic Analysis of 16S (Bacteria/Archaea) and 18S (Eukaryotes) Amplicons

The archaeal and bacterial (16S rRNA gene, V3–V4 region), as well as the eukaryotic (18S rRNA gene, V4–V5 region) hypervariable regions were amplified from the sediment eDNA samples using a nested PCR approach. Locus-specific primers, incorporating Illumina overhang adapter sequences, were utilized to ensure compatibility with subsequent indexing (Table 2).

The first round of PCR was performed in a total volume of 25 µL, containing 2.5 µL of template DNA (~5 ng/µL), 5 µL of each forward and reverse primer (1 µM), and 12.5 µL of 2× KAPA HiFi HotStart ReadyMix (Kapa Biosystems, Wilmington, MA, USA). To maximize the amplification yield from these complex hypersaline sediment matrices, the thermal cycling was increased to 40 cycles (95 °C for 30 s, 55 °C for 30 s, and 72 °C for 30 s). Amplification success and fragment size distribution were verified using the 5300 Fragment Analyzer system (Agilent Technologies, Lawrence, KS, USA) with the Agilent DNF-474 HS NGS Fragment Kit. DNA concentrations were quantified using a Quantus™ Fluorometer (Promega, Madison, WI, USA).

No-Template Controls (NTC) using nuclease-free water were included in each PCR run to detect any potential cross-contamination or primer-dimer formation during the 40-cycle amplification.

The PCR products were purified using AMPure XP beads (Beckman Coulter, Brea, CA, USA) to remove primer dimers and non-specific products.

Subsequent indexing PCR, purification using AMPure XP beads (Beckman Coulter, Brea, CA, USA), and library pooling were performed according to the Illumina MiSeq™ Library Preparation Guide. The final libraries were sequenced on the Illumina MiSeq platform using v3 chemistry (2 × 300 bp paired-end reads).

The Mothur software (v.1.30.2) was used for sequence processing and analysis [75]. Reads were quality-filtered, trimmed, and screened for chimeras using the UCHIME algorithm implemented in Mothur, and detected chimeric sequences were removed. Sequences were then clustered into operational taxonomic units (OTUs) at 97% similarity (0.03 cutoff). For eukaryotic 18S sequences, OTUs were classified using the SILVA v132 eukaryotic 18S database [20], using the naïve Bayesian classifier implemented in Mothur (classify.seqs command) with a bootstrap confidence threshold of 80%. Archaeal sequences were assigned using the non-redundant v138 training set SILVA archaeal and bacterial-specific database to ensure accurate taxonomic resolution [20]. Alpha diversity metrics, including Good’s coverage, Shannon, and Simpson indices, were calculated using Mothur to assess community richness, evenness, and sampling depth for both fungal, bacterial, and archaeal communities.

### 3.4. Nanopore Bacterial 16S rRNA Library Preparation, Sequencing, and Data Analysis

To resolve the taxonomic ambiguities often encountered with short-read sequences, the full-length 16S rRNA gene (~1500 bp) was amplified and sequenced. Libraries were prepared using the 16S Barcoding Kit 24 V14 (SQK-16S114.24) (ONT, Oxford, UK) following the manufacturer’s protocol. Briefly, 16S rRNA genes were amplified using barcoded primers (27F and 1492R equivalents) and LongAmp Taq 2× Master Mix (NEB). The thermal cycling conditions included an initial denaturation at 95 °C for 1 min, 25 cycles of denaturation (95 °C, 20 s), annealing (55 °C, 30 s), and extension (65 °C, 2 min), followed by a final extension at 65 °C for 5 min. Amplicons were purified with AMPure XP beads (0.6× ratio) and quantified using a Quantus™ Fluorometer (Promega). The pooled library (50 fmol) was loaded onto a PromethION Flow Cell (R10.4.1), and the sequencing was performed on a PromethION 2 (P2) Solo device supplied by Oxford Nanopore Technologies (Oxford, UK).

16S rRNA reads were quality filtered, converted to FASTA format, and aligned against the SILVA ribosomal RNA database (release 138.1) using a similarity-based approach implemented in BLAST (version 6.24.20). Taxonomic assignment was then performed using a Lowest Common Ancestor (LCA) algorithm in MEGAN software version 6.24.20 [76], which assigns each OTU to the most specific taxonomic level supported by the sequence data while avoiding over-classification. Assignments were accepted at ≥97% sequence identity for genus-level and ≥99% for species-level classification.

### 3.5. Shotgun Library Preparation, Illumina and Nanopore Sequencing, Taxonomic Profiling, and Functional Annotation

To resolve the functional potential of the microbial communities found in the hypersaline sediments, a hybrid shotgun metagenomic approach was employed, combining the high-accuracy short reads of Illumina with the long-read scaffolds of Oxford Nanopore.

#### 3.5.1. Illumina Library Preparation and NextSeq Sequencing

Metagenomic libraries were prepared using the Illumina DNA Prep Kit (Illumina, San Diego, CA, USA) following the manufacturer’s instructions. This protocol utilizes On-Bead Tagmentation, where bead-linked transposomes simultaneously fragment and tag the metagenomic DNA with adapter sequences. The tagmented DNA was then amplified via a limited-cycle PCR to add dual index adapters. Library quality and fragment size were verified using the 5300 Fragment Analyzer. Sequencing was performed on the Illumina NextSeq™ 500 platform (2 × 150 bp), providing the high-depth coverage required for precise quantitative profiling and error correction of long-read assemblies.

Raw sequencing reads were assessed for quality using *FastQC* http://www.bioinformatics.babraham.ac.uk/projects/fastqc: (accessed on 12 February 2026), including the evaluation of base quality scores, GC content, sequence duplication, and adapter contamination. Illumina reads were then trimmed and filtered with *fastp* https://github.com/opengene/fastp: (accessed on 12 February 2026) [77], to remove low-quality bases and adapters.

#### 3.5.2. Nanopore Native Library Preparation and PromethION Sequencing

Long-read libraries were prepared using the Native Barcoding Kit 24 V14 (SQK-NBD114.24) (ONT, Oxford, UK). This PCR-free method was chosen to avoid amplification bias and to maintain the original representation of the hypersaline microbial community. The workflow followed the manufacturer’s instructions. The final library was sequenced on a PromethION Flow Cell (R10.4.1) using the PromethION 2 (P2) Solo.

Raw Nanopore reads were assessed for quality using *FastQC*, and no stringent trimming or filtering was applied to preserve read length and coverage. Only reads meeting basic quality criteria were retained for downstream analyses, without subsampling, to maintain an accurate quantitative representation of microbial abundance. This method allowed for the retrieval of long genomic fragments, facilitating the identification of complex metabolic gene clusters.

#### 3.5.3. Taxonomic Profiling and Functional Annotation

The taxonomic profiling of shotgun metagenomic data was performed using Kraken2 https://github.com/DerrickWood/kraken2: (accessed on 21 February 2026) [78], followed by abundance re-estimation with Bracken https://github.com/jenniferlu717/Bracken: (accessed on 23 February 2026) [79]. To ensure accuracy, Bracken parameters were adjusted for platform-specific read lengths (100 bp for Illumina and 1000 bp for Nanopore). Functional profiling was conducted using the HUMAnN 3.0 pipeline https://huttenhower.sph.harvard.edu/humann/: (accessed on 23 February 2026), which integrates the UniRef90 protein database for gene family quantification and the MetaCyc database (version 29.1) for metabolic pathway reconstruction. To account for variations in sequencing depth between samples, gene family and pathway abundances were normalized to Copies Per Million (CPM). All analyses were performed using default parameters, except for the high-precision alignment mode enabled for long-read integration. Due to low read counts in the Thyna sample, the shotgun Nanopore data for this site were primarily used for qualitative validation of the dominant functional pathways identified by the deeper Illumina shotgun sequencing.

## 4. Conclusions

This study provides the first comprehensive, multi-platform metagenomic characterization of the Thyna (Sfax) and Abbassia (Kerkennah) solar salterns in Tunisia. By integrating full-length 16S/18S rRNA sequencing with hybrid shotgun metagenomics (Illumina and Nanopore), we highlighted that while both sites share a core extremophilic microbiome, their community structures are distinctively shaped by local environmental filters. The higher salinity at Thyna (170 g/L) fosters a diverse and specialized assembly of extreme halophiles such as *Salinibacter* and *Abyssalbus*, whereas the more moderate conditions at Abbassia (105 g/L) favor halotolerant taxa such as *Salinicoccus* and *Jeotgalicoccus*. Crucially, our methodological comparison revealed that 16S amplicon sequencing significantly overrepresents the *Bacillota* phylum due to ribosomal copy number biases, whereas shotgun metagenomics provides a more balanced and accurate representation of the *Halobacteriota* and *Pseudomonadota* fractions. In conclusion, our results underscore the necessity of using untargeted shotgun approaches to mitigate the inherent biases of amplicon sequencing in extreme environments. This work offers a valuable first glimpse into the ‘dark matter’ of Tunisian salterns, serving as a framework for future high-resolution studies incorporating spatial and temporal replicates to validate the ecological filtering and resilience patterns observed here.

## Figures and Tables

**Table 1:** Physicochemical parameters of the Thyna (Sfax) and Abbassia (Kerkennah) salterns.

Parameter	Thyna (Sfax) Saline Saltern	Abbassia (Kerkennah) Saltern
Latitude/Longitude	34.7200° N/10.7600° E	34.6833° N/11.1167° E
Temperature (°C)	34	36
pH	8	8.2
Salinity (g/L)	170	105
Dissolved Oxygen (mg/L)	3.5	5.25
Electrical Conductivity (mS/cm)	125	120
Turbidity (NTU)	50	75

**Table 2:** Oligonucleotides used as PCR primers to amplify the V3–V4 and V4–V5 hypervariable regions of the archaeal and bacterial 16S rRNA genes, and the eukaryotic 18S rRNA genes.

Primer Name	Oligonucleotide Sequence (5′ → 3′)	Target Region
**Arc340F**	TCGTCGGCAGCGTCAGATGTGTATAAGAGACAGCCCTACGGGGYGCASCAG	Archaeal 16S (V3–V4)
**Arc806R**	GTCTCGTGGGCTCGGAGATGTGTATAAGAGACAGGGACTACVSGGGTATCTAAT	Archaeal 16S (V3–V4)
**16S_fwd**	TCGTCGGCAGCGTCAGATGTGTATAAGAGACAGCCTACGGGNGGCWGCAG	Bacterial 16S (V3–V4)
**16S_rev**	GTCTCGTGGGCTCGGAGATGTGTATAAGAGACAGGACTACHVGGGTATCTAATCC	Bacterial 16S (V3–V4)
**18S_fwd**	TCGTCGGCAGCGTCAGATGTGTATAAGAGACAGCAGCAGCCGCGGTAATTCC	Eukaryotic 18S (V4–V5)
**18S_rev**	GTCTCGTGGGCTCGGAGATGTGTATAAGAGACAGCCCGTGTTGAGTCAAATTAAGC	Eukaryotic 18S (V4–V5)

Note: The underlined nucleotides are the Illumina overhang adapter sequences.

## Data Availability

The data supporting the findings of this study are available within the manuscript and Supplementary Materials. No additional datasets were generated or deposited.

## Supplementary Materials

The following supporting information can be downloaded at: https://www.mdpi.com/article/10.3390/ijms27114714/s1.
