# Supplementary material for: Unraveling the Taxonomic Diversity and Functional Potential of the Tunisian Salterns, Abbassia and Thyna, via Integrated 16S-18S Amplicons and Shotgun Metagenomics
Source: Int J Mol Sci. 2026 May 23;27(11):4714. doi: 10.3390/ijms27114714 (PMC13256939; doi:10.3390/ijms27114714)
Supplement: Supplementary file 1 [file ijms-27-04714-s001.zip › Supplementary_ijms-4263314.R1.pdf]

Supplementary Data

Unraveling the Taxonomic Diversity and Functional Potential of the Tunisian Salterns, Abbassia and Thyna, via Integrated 16S-18S Amplicons and Shotgun Metagenomics

Sondes Mechri [1,2], Afef Najjari [3], Séverine Croze [2], Hadda-Imene Ouzari [3], Marilize Le Roes-Hill [4], Slim Tounsi [5], Joel Lachuer [2,6,*], Bassem Jaouadi [1,*]

[1] *Laboratoire des Biotechnologies Microbiennes et Enzymatiques et Biomolécules (LBMEB), Centre de Biotechnologie de Sfax (CBS), Université de Sfax (USF), Route Sidi Mansour Km 6, BP 1177, Sfax, 3018, Tunisia;*
[2] *Univ Lyon, Université Claude Bernard Lyon 1 (UCBL), Plateforme ProfileXpert de Génomique et Microgénomique, SFR Santé-Lyon-Est, CNRS UMR-S3453, INSERM US7, Faculté de Pharmacie de Lyon, 8 avenue Rockefeller 69373, Lyon Cedex 08, France;*
[3] *Laboratoire Microorganismes et Biomolécules Actives (LMBA), Département de Biologie, Faculté des Sciences de Tunis (FST), Université de Tunis El Manar (UTM), Foyer Universitaire, 20 Rue de Tolède, Tunis 2092, Tunisia;*
[4] *Applied Microbial and Health Biotechnology Institute (AMHBI), Cape Peninsula University of Technology (CPUT), P.O. Box 1906, Bellville 7535, South Africa;*
[5] *Laboratorie de Biopesticides (LB), Centre de Biotechnologie de Sfax (CBS), Université de Sfax (USF), Route Sidi Mansour Km 6, BP 1177, Sfax, 3018, Tunisia;*
[6] *Univ Lyon, Université Claude Bernard Lyon 1 (UCBL), UMR INSERM 1052, CNRS 5286, Centre Léon Bérard, Centre de Recherche en Cancérologie de Lyon (CRCL), 28 Rue Laennec, 69008 Lyon Cedex 08, France*

* Correspondence: joel.lachuer@univ-lyon1.fr; bassem.jaouadi@cbs.rnrt.tn

Table S1: DNA purity ratios ($A_{260/280}$ and $A_{260/230}$) obtained by Implen™ Nanophotometer™ analyses of the environmental DNA isolated from the two sampling sites, the Thyna and Abbassia salterns, when using three different commercial DNA isolation kits.

Extraction Kit	Sample	$A_{260/280}$	$A_{260/230}$
DNeasy PowerMax Soil kit	Thyna	1.89	1.99
	Abbassia	1.97	2.04
ZymoBIOMICS™ 96 MagBead kit	Thyna	2.01	2.31
	Abbassia	1.93	2.06
Quick-DNA Fecal/Soil kit	Thyna	1.73	2.18
	Abbassia	1.94	2.06

--

Table S2: DNA yields (concentration) of the environmental DNA isolated from the Thyna and Abbassia saltern samples, using three different commercial DNA extraction kits and as determined by the use of a Quantus™ Fluorometer.

Extraction Kit	Sample	Volume (μL)	Final Conc. (ng/μL)	Total eDNA (ng)
DNeasy PowerMax Soil kit	Thyna	100	45.2	4520
	Abbassia	100	32.1	3210
ZymoBIOMICS™ 96 MagBead kit	Thyna	50	52.4	2620
	Abbassia	50	40.8	2040
Quick-DNA Fecal/Soil kit	Thyna	50	58.2	2910
	Abbassia	50	65.4	3270

A

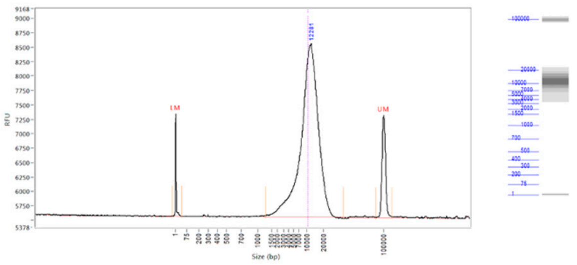

Peak	Size (bp)	Conc. (ng/uL)	From (bp)	To (bp)	Avg. Size (bp)	CV%	RFU	Corr. Peak Area
1	1 (LM)	0.3165	0	41	1	365.81	1775	10.716
2	12281	94.7301	1274	46034	11273	41.76	3009	267.255
3	100000 (UM)	2.4483	>60000	>60000	>60000	2.37	1781	82.886

B

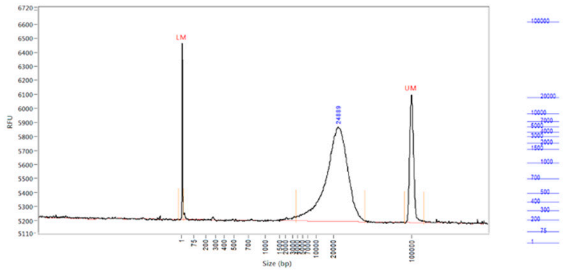

Peak	Size (bp)	Conc. (ng/uL)	From (bp)	To (bp)	Avg. Size (bp)	CV%	RFU	Corr. Peak Area
1	1 (LM)	0.2710	0	9	0	-975.20	1254	7.317
2	24889	25.2377	3656	51935	23096	39.78	673	56.779
3	100000 (UM)	0.3999	93320	112215	100186	2.21	909	10.797

C

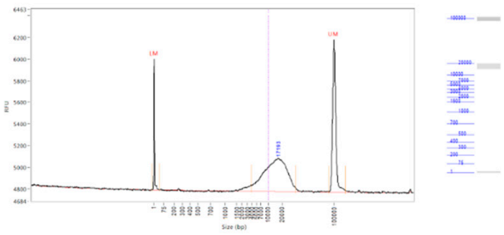

Peak	Size (bp)	Conc. (ng/uL)	From (bp)	To (bp)	Avg. Size (bp)	CV%	RFU	Corr. Peak Area
1	1 (LM)	0.3165	0	42	1	334.24	1212	7.397
2	17193	25.5618	3912	41473	15887	49.80	306	49.780
3	100000 (UM)	2.9449	>60000	>60000	>60000	3.27	1410	68.820

D

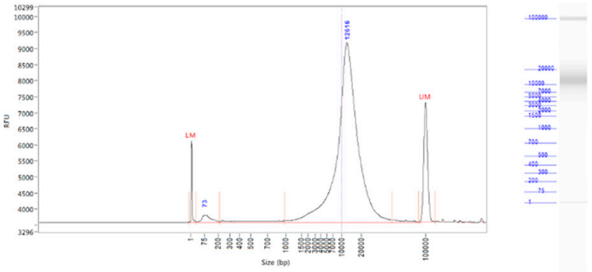

Peak	Size (bp)	Conc. (ng/uL)	From (bp)	To (bp)	Avg. Size (bp)	CV%	RFU	Corr. Peak Area
1	1 (LM)	0.2490	0	27	1	376.98	2532	15.599
2	73	2.7430	27	210	96	40.23	227	14.323
3	12616	107.5038	978	58539	13353	55.72	5590	561.325
4	100000 (UM)	1.3860	>60000	>60000	>60000	2.36	3723	86.843

E

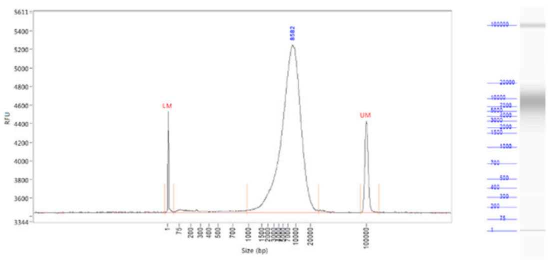

Peak	Size (bp)	Conc. (ng/uL)	From (bp)	To (bp)	Avg. Size (bp)	CV%	RFU	Corr. Peak Area
1	1 (LM)	0.3184	0	38	2	394.01	1087	6.985
2	8582	86.0196	977	31344	8162	50.39	1806	157.240
3	100000 (UM)	0.5313	91642	118891	100388	2.69	987	11.655

F

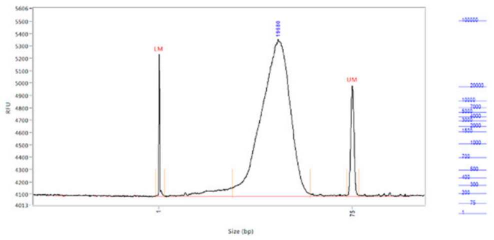

Peak	Size (bp)	Conc. (ng/uL)	From (bp)	To (bp)	Avg. Size (bp)	CV%	RFU	Corr. Peak Area
1	1 (LM)	Inf	0	10434	3145	148.23	1151	7.121
2	19680	Inf	15969	31796	19961	11.81	1268	174.261
3	100000 (UM)	Inf	46790	51927	49123	1.31	894	10.275

Figure S1: DNA integrity and size distribution profiles from the 5300 Fragment Analyzer system (Agilent). Electropherograms (A-B) represent the DNeasy PowerMax Soil kit, (C-D) the ZymoBIOMICS 96 MagBead kit, and (E-F) the Quick-DNA Fecal/Soil kit for Thyna and Abbassia samples, respectively. The X-axis represents the fragment size (bp) and the Y-axis represents fluorescence intensity (RFU). The presence of a prominent peak in the high-molecular-weight region confirms the suitability of the extracted DNA for both Illumina shotgun and Oxford Nanopore long-read sequencing.

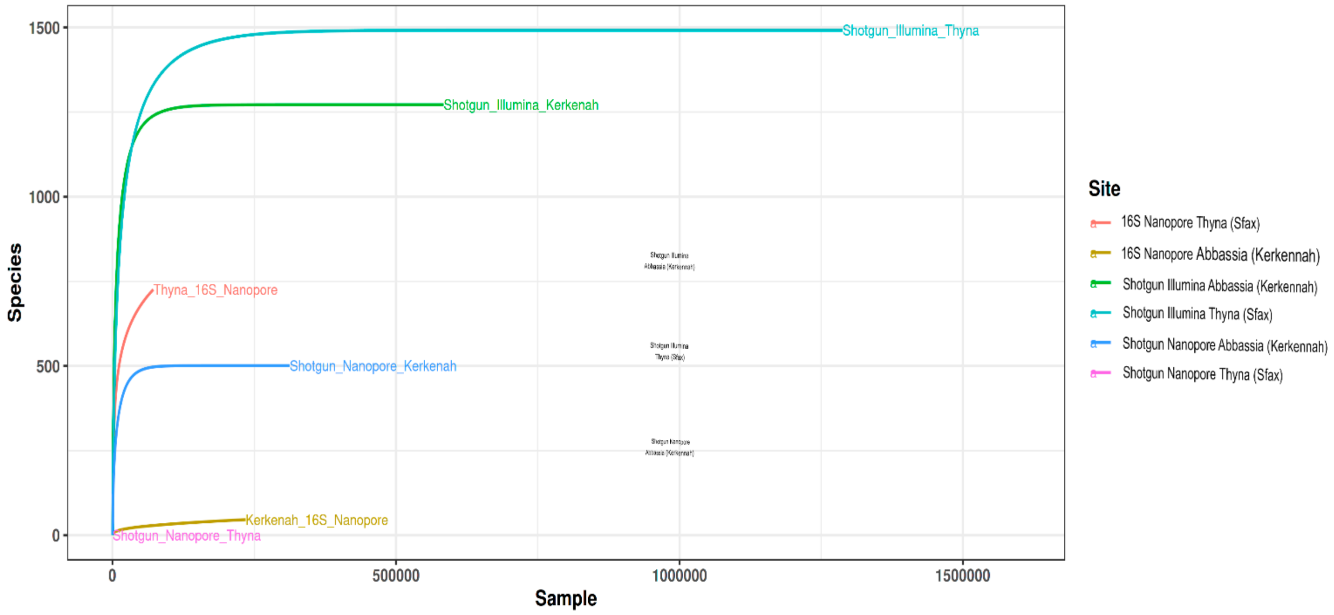

Figure S2: Rarefaction curves showing species richness across six sequence data sets.
